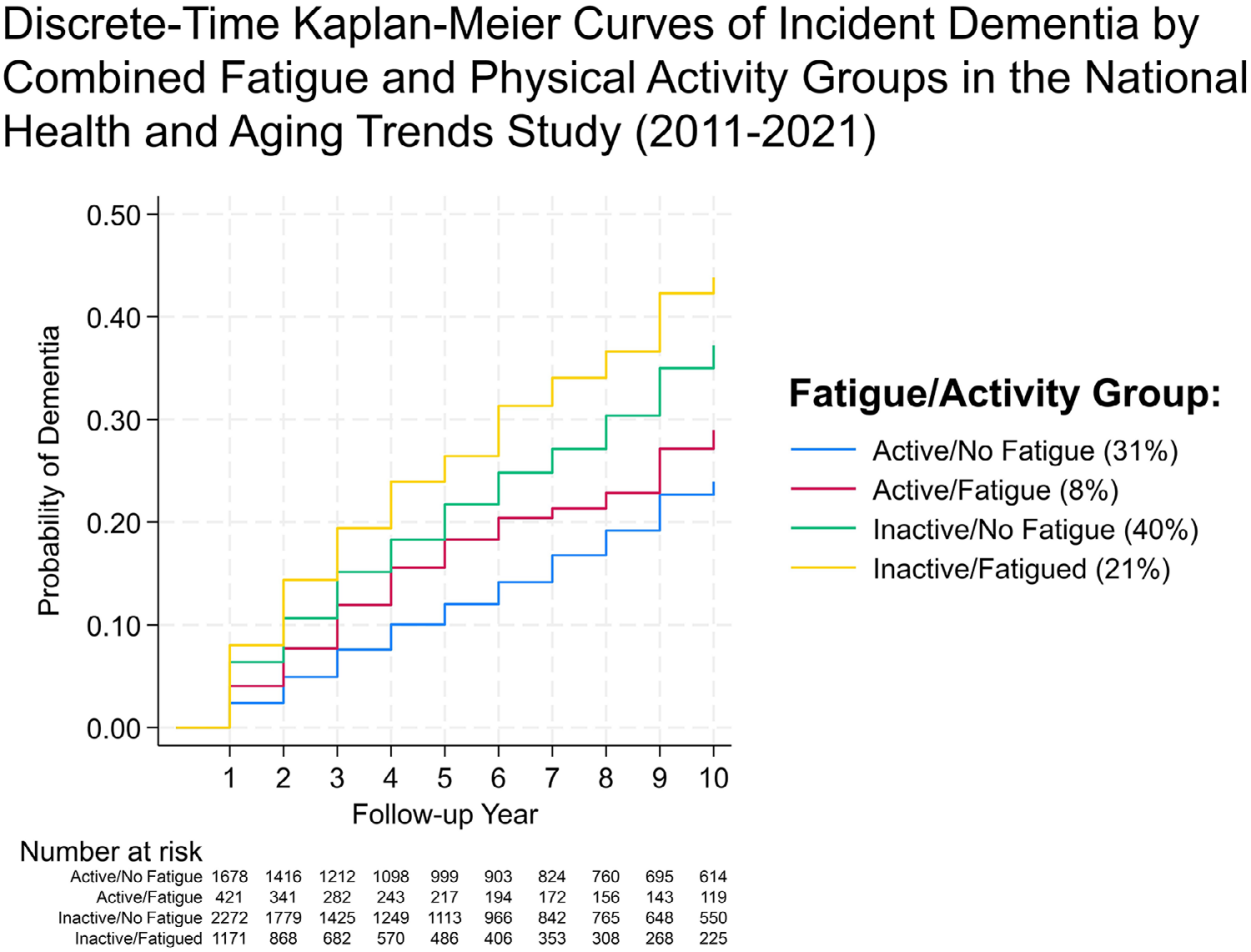# Fatigue predicts 10‐year risk of incident dementia in a population representative cohort of United States Medicare beneficiaries

**DOI:** 10.1002/alz.085785

**Published:** 2025-01-09

**Authors:** Kyle D. Moored, Nancy W. Glynn, Michelle C Carlson, Patrick T. Donahue, Jennifer A Schrack

**Affiliations:** ^1^ Johns Hopkins Bloomberg School of Public Health, Baltimore, MD USA; ^2^ University of Pittsburgh, Pittsburgh, PA USA

## Abstract

**Background:**

Fatigue is prevalent in later life and may increase dementia risk independent of health conditions. Yet, existing epidemiologic studies include samples that are not nationally representative of U.S. older adults. These studies have also not examined fatigue in combination with current physical activity level, which is important because higher fatigue ratings may also stem from a more active lifestyle that could lower dementia risk. We examined whether self‐reported fatigue, both independently and jointly with self‐reported physical activity, was associated with 10‐year risk of incident dementia in U.S. older adults.

**Method:**

Participants were from the National Health and Aging Trends Study (2011‐2021, N = 5,167), a population representative cohort of U.S. Medicare beneficiaries. Fatigue was self‐reported as having low energy in the past month that interfered with activities (yes/no). Participants also reported past‐month engagement in any vigorous activity that increased their heart rate (e.g., working out; yes/no). Probable dementia cases were ascertained using cognitive testing and proxy reporting. Analyses included discrete‐time proportional hazards models with sample weighting to account for survey design and adjusted for baseline age, sex, race/ethnicity, education, BMI, number of health conditions, smoking status, social isolation, and depressive symptoms.

**Result:**

Twenty‐nine percent of participants reported fatigue. Frequencies of combined fatigue and activity groups included: “active/no fatigue” (31%), “active/fatigued” (8%), “inactive/no fatigue” (40%), “inactive/fatigued” (21%). There were 1,089 incident cases of probable dementia for up to 10 years of follow‐up (M±SD = 5.4±3.6 years). Individuals reporting fatigue (vs. no fatigue) had a 16% greater risk of incident dementia after full covariate adjustment (HR = 1.16, 95% CI: 1.01, 1.34, p = .041). Compared to the “active/no fatigue” group, the “inactive/fatigued” group had a 40% greater risk of incident dementia after adjustment (HR = 1.40, 95% CI: 1.16, 1.69, p = .001), but risk did not significantly differ for “active/fatigued” and “inactive/no fatigue” groups (p’s>.05).

**Conclusion:**

Fatigue may be a signal of increased risk of incident dementia in U.S. older adults, independent of depressive symptoms and other health characteristics. Individuals reporting both fatigue and inactivity had the highest risk of dementia, suggesting that fatigue may be a symptom of underlying pathological processes, especially among those with low activity levels.